# Volumetric associations between uncinate fasciculus, amygdala, and trait anxiety

**DOI:** 10.1186/1471-2202-13-4

**Published:** 2012-01-04

**Authors:** Volker Baur, Jürgen Hänggi, Lutz Jäncke

**Affiliations:** 1 Division Neuropsychology, Institute of Psychology, University of Zurich, Switzerland; 2 Center for Integrative Human Physiology, University of Zurich, Switzerland; 3 International Normal Aging and Plasticity Imaging Center (INAPIC), University of Zurich, Switzerland

**Keywords:** trait anxiety, uncinate fasciculus, amygdala, hippocampus, volume, white matter, grey matter, tractography, diffusion tensor imaging, subcortical segmentation

## Abstract

**Background:**

Recent investigations of white matter (WM) connectivity suggest an important role of the uncinate fasciculus (UF), connecting anterior temporal areas including the amygdala with prefrontal-/orbitofrontal cortices, for anxiety-related processes. Volume of the UF, however, has rarely been investigated, but may be an important measure of structural connectivity underlying limbic neuronal circuits associated with anxiety. Since UF volumetric measures are newly applied measures, it is necessary to cross-validate them using further neural and behavioral indicators of anxiety.

**Results:**

In a group of 32 subjects not reporting any history of psychiatric disorders, we identified a negative correlation between left UF volume and trait anxiety, a finding that is in line with previous results. On the other hand, volume of the left amygdala, which is strongly connected with the UF, was positively correlated with trait anxiety. In addition, volumes of the left UF and left amygdala were inversely associated.

**Conclusions:**

The present study emphasizes the role of the left UF as candidate WM fiber bundle associated with anxiety-related processes and suggests that fiber bundle volume is a WM measure of particular interest. Moreover, these results substantiate the structural relatedness of UF and amygdala by a non-invasive imaging method. The UF-amygdala complex may be pivotal for the control of trait anxiety.

## Background

A growing body of neuroimaging studies links white matter (WM) measures to anxiety-related psychological processes [1-4]. These studies in summary point to the uncinate fasciculus (UF), a prominent fronto-temporal fiber tract known to innervate the amygdala [5-8]. The amygdala has been shown to be part of a limbic network that is hyper-responsive in individuals with increased anxiety [9] and in patients with anxiety disorders [10]. In this network, the UF is a pivotal part providing a link from the amygdala to prefrontal/orbitofrontal cortical areas, and thus is involved in modulating anxiety [11]. Therefore, the UF is of particular interest for investigating the relation between anxiety and WM morphometry.

**Figure 1:**
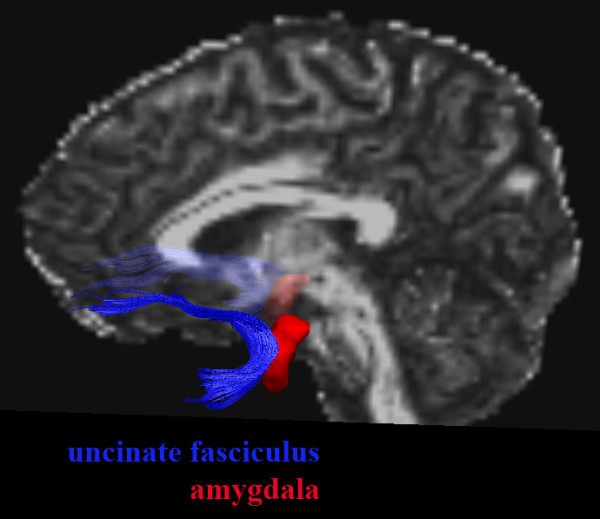
**Topography of the uncinate fasciculus and amygdala**. Example of their relative locations, shown for one subject in native space, lateral view onto the left hemisphere (right hemisphere on background).

Using fiber tractography, we previously showed that patients with social anxiety disorder compared to healthy subjects demonstrate reduced volume of the left UF, suggesting fronto-temporal structural hypoconnectivity [12]. Regarding the preliminary nature of this result, identification of correlates of UF volume in a completely independent study sample is needed to validate UF volumetric measure and to further characterize in what way left UF volume is associated with anxiety. Anxiety is complex, involving emotional, cognitive, motivational, and physiological components and is dimensional with high inter-individual variability [13]. Personality traits are seen as intra-individual stable factors, which can be linked to brain structure (e.g., [14,15]). *Trait anxiety *is defined as a psychological construct including several components, which merge and result in feelings such as discomfort, nervousness, and unpleasantness. Trait anxiety is a fixed stage of anxiety existing for a relative long duration or is even stable over a longer time period. Strong trait anxiety is also seen as the propensity to become extra-anxious in the context of provoking stimuli [13]. Here, we sought to explore the dimensional association of UF volume with trait anxiety as assessed by means of the widely used Spielberger State-Trait Anxiety Inventory (STAI) [13]. In addition to WM, we also investigated grey matter (GM) volumes of subcortical structures. The amygdala-hippocampus complex has been implicated in fear- and anxiety-related behavior [16]. Functional imaging studies have also demonstrated a link between individual differences in trait anxiety and amygdala activity by showing increased amygdala activity with increasing anxiety [9,17-19]. Regarding volumetric associations with trait anxiety, two studies point to a prominent role of the left amygdala compared to the other subcortical structures [20,21]. However, studies focusing on the specific anxiety-related neuroanatomical features of the amygdala and linked pathways are still rare. In addition, these studies do not consistently report anxiety-related volumetric associations for the amygdala (see also [22]). Thus, we focus - beside the UF - on the amygdala GM volume and its relatedness to anxiety as well as its relatedness to UF volume (for the topography of UF and amygdala, see figure 1).

In the present study, we investigated volumes of the UF, the amygdala, and the hippocampus in a non-clinical sample of 32 subjects with different levels of trait anxiety. For this study, we formulated the following hypotheses:

(i) UF volume should correlate negatively with trait anxiety (according to the previous finding of reduced left UF volume in social anxiety disorder [12]);

(ii) The amygdala volume should correlate positively or negatively with trait anxiety;

(iii) UF and amygdala volumes should be strongly intercorrelated either negatively or positively, depending also on the association between trait anxiety and amygdala volume;

Since the anterior hippocampus has been associated with anxiety and this hippocampal part is also tightly linked to the amygdala (amygdala-hippocampus complex) [16], we also anticipated a correlation between trait anxiety and hippocampus volume.

## Results

### Demographic, psychometric and global anatomical measures

Demographic and psychometric measures are summarized in table 1. STAI values ranged from 25 to 55 (see additional file 1, figure S1). Mean (standard deviation) intra-cranial volume was 1076.6 (118.8) ml. Intra-cranial volume showed significant positive associations (assessed by Pearson bivariate correlation) with volumes of left UF (*r *= 0.52, *p *< 0.01), right UF (*r *= 0.61, *p *< 0.001), left amygdala (*r *= 0.39, *p *< 0.05), and left hippocampus (*r *= 0.46, *p *< 0.01); non-significant positive associations were observed for right amygdala (*r *= 0.20, *p *= 0.27) and right hippocampus (*r *= 0.26, *p *= 0.15). There was no correlation of intra-cranial volume with trait anxiety (*r *= 0.08, *p *= 0.68). For all further statistical analyses, relative values were used for each measure (that is, local WM/GM volume divided by global, intra-cranial volume).

**Table 1:** Demographic and psychometric measures (*n *= 32)

	mean (SD)	range	association with trait anxiety ^a^
age (years)	24.9 (4.6)	20-37	-0.26 (0.159)
education (years)	16.1 (2.9)	12-22	0.07 (0.688)

trait anxiety ^b^	39.3 (8.8)	25-55	
depression ^c^	5.0 (4.3)	0-16	0.59 (< 0.001)
anxiety sensitivity ^d^	19.0 (8.1)	5-37	0.36 (0.042)
behavioral inhibition ^e^	23.8 (5.3)	14-36	0.66 (< 0.001)
neuroticism ^f^	9.5 (4.6)	2-20	0.74 (< 0.001)
extraversion ^g^	11.3 (3.7)	5-19	-0.23 (0.209)

^a ^using Pearson bivariate correlations, shown are *r*-values (*p*-values in brackets)

^b ^according to the Spielberger State-Trait Anxiety Inventory (STAI), trait section

^c ^according to the Beck Depression Inventory (BDI)

^d ^according to the Anxiety Sensitivity Index 3 (ASI-3)

^e ^according to the BIS subscale of the Action Regulating Emotional Systems (ARES) questionnaire

^f ^according to the Eysenck Personality Inventory (EPI), neuroticism subscale

^g ^according to the Eysenck Personality Inventory (EPI), extraversion subscale

### Associations of trait anxiety with white matter and grey matter volumes

**Figure 2:**
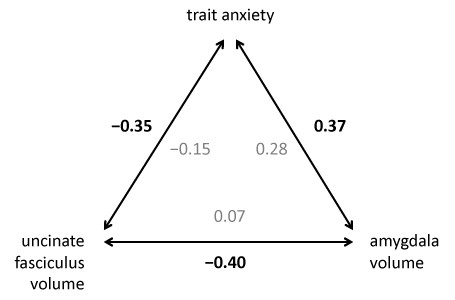
**Triangular association between trait anxiety, uncinate fasciculus volume, and amygdala volume**. Bold (outer) values indicate significant partial correlations (controlling for age, sex, and depression) for the left hemisphere; grey (inner) values indicate respective non-significant correlations for the right hemisphere.

Correlations are summarized in figure 2.

*Left hemisphere*: Trait anxiety was negatively correlated with UF volume (*r *= -0.35, *p *= 0.03), but not with volume of the inferior fronto-occipital fasciculus (*r *= 0.01, *p *= 0.94). Regarding subcortical GM, trait anxiety was positively correlated with amygdala volume (*r *= 0.37, *p *= 0.048). The correlation for the hippocampus was marginally significant, conversely, there was no significant correlation for the caudate nucleus (see table 2). UF volume correlated negatively with amygdala volume (*r *= -0.40, *p *= 0.03), but not significantly with the hippocampus or caudate nucleus (see table 3). Scatter plots are shown in additional file 1, figure S2. Importantly, results did not change qualitatively when excluding the left-handed subject and the three subjects reporting a history of psychotherapeutic treatment. Correlations of fractional anisotropy, a measure indicative of fiber organization and integrity, for the left UF with trait anxiety and amygdala volume as well as UF volume are presented in additional file 1, table S1. These correlations were similar to those observed for volume.

**Table 2:** Associations between white matter/grey matter volumes and trait anxiety

hemisphere	brain tissue	structure	association with trait anxiety ^a^
left	*white matter*	uncinate fasciculus	**-0.35 (0.030) **^b^
		inferior fronto-occipital fasciculus	0.01 (0.939)
	
	*grey matter*	amygdala	**0.37 (0.048)**
		hippocampus	*0.34 (0.075)*
		caudate	0.09 (0.639)

right	*white matter*	uncinate fasciculus	-0.15 (0.449)
		inferior fronto-occipital fasciculus	-0.04 (0.821)
	
	*grey matter*	amygdala	0.28 (0.144)
		hippocampus	**0.44 (0.018)**
		caudate	0.07 (0.710)

^a ^using partial correlations with age, sex, and depression as covariates of no interest, shown are *r*-values (*p*-values in brackets, significant results indicated in bold, marginally significant results indicated in italics). Note: For uncinate fasciculus, amygdala, and hippocampus, results underlay hypothesis-driven tests; the remaining tests underlay the null hypothesis.

^b ^one-tailed

**Table 3:** Associations between white matter and subcortical grey matter volumes ^a^

			*white matter*			
			left		right	
			
			UF	IFOF	UF	IFOF
*grey matter*	left	amygdala	**-0.40**	0.24		
		hippocampus	0.01	0.20		
		caudate	-0.16	0.11		
	
	right	amygdala			0.07	0.25
		hippocampus			0.12	0.17
		caudate			*-0.32*	-0.05

^a ^using partial correlations with age, sex, and depression as covariates of no interest, shown are *r*-values (significant correlations at *p *< 0.05 indicated in bold, marginally significant correlations at *p *< 0.10 indicated in italics)

UF: uncinate fasciculus, IFOF: inferior fronto-occipital fasciculus

Note: Significant/marginally significant results correspond to uncorrected *p*-values. Except of for UF-amygdala volumetric associations, tests underlay the null hypothesis.

*Right hemisphere*: Except for the hippocampus, no significant correlations of WM or GM volumes with trait anxiety (see table 2) were observed. Moreover, there was no UF-amygdala volumetric association in the right hemisphere (see table 3).

To test discriminant validity of associations with trait anxiety, we additionally assessed correlations with depression, anxiety sensitivity, behavioral inhibition, and neuroticism for each WM/GM volume of interest on an explorative level. Here, no associations were found in both hemispheres (shown in additional file 1, table S2). Post-hoc exploratory tests did not show significant correlations with trait anxiety for the remaining subcortical volumes (additional file 1, table S3). Absolute and relative volumes of all examined WM and GM structures are listed in additional file 1, table S4.

## Discussion

This study's focus was on the associations between anxiety and the volumes of the UF, amygdala, and hippocampus as well as on the associations between UF and amygdala. In our non-clinical sample, trait anxiety correlated negatively with UF volume and positively with amygdala volume. In addition, the volumes of UF and amygdala were inversely correlated with each other. These effects were prominent in the left hemisphere and independent of global brain volume, age, sex, and current depression.

In a previous study of our group, we demonstrated reduced left UF volume in patients suffering from social anxiety disorder compared to healthy control subjects using the same deterministic tractography approach [12]. The main goal of the present study was to assess the relation between UF volume and trait anxiety in a non-clinical sample. In this particular sample, we identified a negative correlation of the left UF volume with trait anxiety. In our previous study where we examined a clinical sample, we demonstrated reduced left UF volume in pathologically anxious patients [12]. Both studies demonstrate that anxiety is negatively related to left UF volume, with high anxiety associated with smaller UF volumes. Thus, both studies using entirely different samples point into the same direction and emphasize the pivotal role of the left UF volume in the modulation of anxiety. What may the negative correlation between UF volume and trait anxiety point to? The left UF volume might indicate the structural prerequisites for the efficiency of left amygdala-orbitofrontal functional interactions. These interactions underlie reappraisal as an emotion regulation process (e.g. [23]) and are critical for reappraisal-initiated reduction of negative affects [24]. Moreover, changed cross-correlated hemodynamic responses in amygdala-orbitofrontal areas have been demonstrated in pathological anxiety [25,26]. Thus, our result supports the notion that specific features of structural and functional connectivity within the amygdala-orbitofrontal network are crucial in modulating and possibly determining individual anxiety [11]. The UF may be considered the main facilitator of signal propagation from and to the amygdala in interactions with the orbitofrontal cortex. It is a "limbic" tract [27,28] and, in fact, has been described in post-mortem and non-human primate studies to innervate the amygdala [5-7]. Yet, further areas also interlinked by the UF may be of interest as well with respect to the present results. The anterior temporal cortex/temporal pole has been related to anxiety [29] and might serve as a relay station between frontal cortex and amygdala, given also strong (possibly UF-independent) projections from the temporal pole to the amygdala [30,31]. The present approach used deterministic tractography of well-characterized fiber bundles according to standardized protocols, encompassing the UF *as a whole*. Thus, specific subparts of the UF could not be delineated with this approach. Further studies using more sophisticated approaches could try to disentangle UF fiber subbundles connecting the amygdala and the orbitofrontal cortex [31,32]. As a supplemental and commonly assessed measure, fractional anisotropy of the left UF was similarly correlated with trait anxiety as compared to volume (additional file 1, table S1). In addition, UF volume and fractional anisotropy were directly positively correlated. It was, however, not within the scope of the present study to explicitly contrast these two measures of WM connectivity. Taken together, the present data rather suggest that UF volume is a measure of interest beside fractional anisotropy.

A further goal of the present study was to examine whether the left UF volume correlates with volume measures of adjacently located brain areas in subcortical GM. In this context we identified, to the best of our knowledge for the first time, that the left-sided UF volume negatively correlated with the left-sided amygdala volume. Beside the relationship between left UF volume and trait anxiety, we also identified a significant relationship between left amygdala volume and trait anxiety. In addition to our study, there are three studies that have examined the relationship between amygdala volume and trait anxiety so far, with different findings. Two of them reported negative correlations [20,21], one no correlation [22], and our study reports a positive correlation. The reason for these discrepant findings is currently difficult to disentangle due to sample (age etc.) and methodological differences (voxel-based morphometry vs. automatic subcortical segmentation). Further studies are needed to examine the influences that are modulating the amygdala-anxiety relationship. From an intuitive point of view, a positive correlation between amygdala volume and trait anxiety may be plausible: Arguing that a larger amygdala in strong trait-anxious subjects may reflect increased usage would be in line with a couple of studies showing a positive association between amygdala activation and trait anxiety [9,17-19]. Beside the amygdala, the right hippocampus was significantly and the left hippocampus was trend-wise positively correlated with trait anxiety in the present study (see also [33]). The anterior hippocampus has been implicated in anxiety-related behavior [16], as it is also closely connected to the amygdala. As such, our results support the view that the amygdala-hippocampus *complex *is linked to trait anxiety.

The reasons for these specific volumetric associations of the UF, amygdala and hippocampus with trait anxiety as well as between UF and amygdala are unclear so far. However, two possible interpretations can be offered here: First, it could be that the specific structural features we uncovered here are the anatomical prerequisites for inefficient and hyper-responsive anxiety behavior in these subjects. In other words, if this explanation is true, the extent of anxiety-related reactions relies on the specific anatomical features of the UF-amygdala complex. The second interpretation relates more to experience-related structural modifications of the UF-amygdala complex. It could be that in high anxious subjects, the amygdala is more often activated, at the end causing a use-dependent increase of the amygdala volume. Due to this increase, the preponderance to automatically react with anxious behavior increases with less influence from the frontal cortex, causing a decrease of UF volume. Which of these explanations hold true cannot be determined with this experimental design. Longitudinal studies are needed to decide whether a kind of genetically/biologically defined anatomical prerequisite results in anxious reactions or whether frequent fear and anxious feelings modify the structure of involved brain areas.

The results of the present study may also raise the question of laterality with regard to volumetric associations between UF, amygdala, and trait anxiety. The fact that these associations were prominent in the left hemisphere may prompt further investigations to directly address the issue of a possible lateralized involvement of these volumes implicated in limbic emotional circuits associated with anxiety.

### Limitations

A major finding of this study is based on self-report data by the participants and it cannot be excluded that there are biases in terms of social desirability. However, the STAI is widely used to measure trait anxiety and there is currently no objective method available to quantify trait anxiety. Due to the reliability of around 0.8 of the STAI [13] and the assumed reliability of our anatomical measurements of approximately 0.9 [12], a maximum structure-behavior relationship of *r *_structure-anxiety, measured _= *r *_structure-anxiety, real _* sqrt(0.8*0.9) [34] can principally be obtained with the applied methods. Thus we are pretty sure that the captured correlations are in the range one could anticipate.

A further limitation is the cross-sectional nature of this study. Thus, we are not in the position to explain our findings being due to experience-driven or biologically-/genetically-driven influences. Longitudinal studies are thus needed to examine the development of anxiety-structure relationships more precisely.

## Conclusions

The left-sided UF-amygdala complex is strongly related to anxiety, even in non-pathological, non-clinical subjects. This study is in line with a previous study that has identified exactly the same quality of relationship between UF anatomical features and anxiety in a pathologically anxious sample. Taken together, these and other studies support the notion that the UF-amygdala complex is pivotal for the control of trait anxiety.

## Methods

### Subjects

We called attention to the study by advertisement on mailing lists and notice boards in the University Zurich buildings. In the first part of this project, 218 subjects took part at an online-screening test where they completed the STAI (trait section) [13] and specified socio-demographic characteristics. In addition, they were asked with respect to exclusion criteria, which were general contraindications against magnetic resonance imaging (MRI), consummation of drugs, excessive consummation of alcohol and nicotine, medication affecting the central nervous system, known history of neurologic or psychiatric disorders, pregnancy, and age over 40. Subjects were selected based on their anxiety levels assessed in the online-screening in order to achieve an equal distribution of trait anxiety in the MRI study group. Finally, 35 healthy subjects (18 female, 17 male) were asked and were willing to participate in the MRI study. Absence of exclusion criteria was confirmed for each subject prior to scanning. None of the subjects reported any current or past neurologic and psychiatric disorders. Three subjects reported a history of psychotherapeutic treatment. None of the subjects reported any current medication. One participant reported a history of antidepressant medication. This participant was excluded from the study. To determine discriminant validity of associations with trait anxiety, further affect-related traits were assessed in addition to the STAI. Before scanning, subjects completed the Anxiety Sensitivity Index questionnaire (ASI-3) [35], Beck Depression Inventory (BDI) [36], Action Regulating Emotion Systems questionnaire (ARES) [37] and Eysenck Personality Inventory (EPI, Form A) [38]. The EPI contains neuroticism and extraversion subscales as well as a "lie" scale (EPI-L). The EPI-L consists of nine items (range: 0-9) assessing response behavior indicative of social desirability (retest-reliability: 0.71, external validity: 0.64) [39]. Since the results of the current study substantially relied on trait anxiety, which was assessed as self-report based on subjective appraisal by the participant, biases in terms of social desirability cannot be ruled out [13]. It has been shown that a group of subjects declaring low anxiety is heterogeneous in terms of social desirability proneness, which biases the results when comparing with high anxious subjects [40]. We aimed at identifying those participants who may be prone to social desirable response behavior, indicated by a high score on the EPI-L. Thus, all participants with values of 7 and higher on the EPI-L (corresponding to the upper third of the theoretical range of the scale) were not considered for statistical analysis. Applying this criterion, two subjects (with values of 7 and 8, respectively) were excluded. Of note, these participants had STAI values of 29 and 22, respectively, thus declaring low trait anxiety on the STAI (theoretical range of the STAI: 20-80). Mean (standard deviation) "lie" score of the remaining subjects was 2.8 (1.5). The final sample consisted of 32 subjects (18 female, 14 male). One person was left-handed, all other were right-handed according to self-report and the Annett questionnaire [41]. Written informed consent was obtained from all participants. Subjects were compensated for their participation. The study was approved by the cantonal ethics committee (Zurich) and conforms to the Helsinki Declaration.

### Magnetic resonance imaging data acquisition

For each participant, one diffusion- and one T1-weighted scan were obtained. Scans were acquired on a 3-T Philips Ingenia whole-body scanner (Philips Medical Systems, Best, The Netherlands) equipped with a transmit-receive body coil and a commercial 15-element sensitivity encoding (SENSE) head coil array.

One diffusion-weighted spin-echo echo-planar imaging (EPI) sequence was applied with a spatial resolution of 2.0 × 2.0 × 2.0 mm^3 ^(matrix: 112 × 112 pixels, 75 slices in transversal plane). Further imaging parameters were: field of view, 224 × 224 mm^2^; echo time, 63.1 ms; repetition time, 18,941.2 ms; flip-angle, 90°; SENSE factor, 2. Diffusion was measured along 64 non-collinear directions (*b *= 1000 s/mm^2^) preceded by a non-diffusion-weighted volume (reference volume, *b *= 0 s/mm^2^). Scan time was about 23 minutes.

One volumetric 3D T1-weighted gradient echo sequence (fast field echo) with a spatial resolution of 0.94 × 0.94 × 1.00 mm^3 ^(matrix: 256 × 256 pixels, 160 slices in sagittal plane) was applied. Further imaging parameters were: field of view, 240 × 240 mm^2^; echo time, 3.7 ms; repetition time, 8.08 ms; flip-angle, 90°; SENSE factor, 1.5. Scan time was about 8 minutes.

### Diffusion tensor imaging preprocessing and tractography

Preprocessing was done with FMRIB Software Library (FSL) Version 4.1.8 [42]http://www.fmrib.ox.ac.uk/fsl, further processing and deterministic tractography was done using Diffusion Toolkit 0.6.1 and TrackVis 0.5.1 [43]http://www.trackvis.org. Preprocessing and manual tractography were performed exactly as described previously [12] (shown also in additional file 2, methods S1). Tractography was performed for the UF and the inferior fronto-occipital fasciculus. The inferior fronto-occipital fasciculus was considered suitable as control tract because it shares a common trajectory with the UF in the frontal lobe [28]. As being part of the well-characterized, large association fiber bundles, manual tractography of the UF and inferior fronto-occipital fasciculus is feasible in a standardized manner [44]. High inter-rater reliability has been demonstrated for both tracts [12,45].

### Segmentation of subcortical structures

Volumetric segmentation was performed using the FreeSurfer image analysis suite (version 5.1.0; surfer.nmr.mgh.harvard.edu). A detailed description is provided in additional file 2, methods S2. Volumes of subcortical structures (amygdala and hippocampus as structures of interest; caudate nucleus as control structure) as well as total intra-cranial volume were extracted for each participant.

### Statistical analysis

To control for global volume (total intra-cranial volume), local WM/GM volume was divided by global, intra-cranial volume. As such, relative volume values were obtained for each measure of interest and used for statistical analysis. For our main approach of *dimensional *associations between trait anxiety (as assessed by the STAI) and WM/GM volume, partial correlations were computed including age, sex, and current depression (as assessed by the BDI) as covariates of no interest, using IBM SPSS Statistics (version 19, SPSS Inc, an IBM company, Armonk, NY). The significance level was α = 0.05. All *p*-values are reported uncorrected. We applied strictly hypothesis-driven tests for associations between trait anxiety, UF volume, and amygdala/hippocampus volume. The remaining tests referred to WM/GM control structures and to psychometric variables other than trait anxiety to assess discriminant validity. These tests relied on the assumption *not *to detect a significant correlation. Thus, we refrained from correcting for multiple comparisons to avoid Type II error [46]. For the association between trait anxiety and left UF volume, *p*-value was interpreted one-tailed because of a directional a-priori hypothesis due to previous evidence [12]. All other *p*-values were two-tailed (given non-directional hypotheses).

## Competing interests

The authors declare that they have no competing interests.

## Authors' contributions

VB, JH and LJ designed the study. VB, JH and LJ undertook the statistical analysis. VB wrote the first draft of the manuscript. All authors approved the final manuscript.

## Supplementary Material

Additional file 1 **Supplemental Results**. Distribution of trait anxiety across subjects (figure S1); Scatter plots for associations between trait anxiety and white matter/grey matter volumes of interest (figure S2); Associations of mean fractional anisotropy across the uncinate fasciculus with trait anxiety and amygdala volume (table S1); Associations of uncinate fasciculus, amygdala and hippocampus volume with depression, anxiety sensitivity, behavioral inhibition, and neuroticism (table S2); Associations between the remaining grey matter volumes and trait anxiety (table S3); Absolute and relative volumes of all examined white matter and grey matter structures (table S4). Click here for file

Additional file 2 **Supplemental Methods**. Diffusion tensor imaging data preprocessing and tractography (methods S1); Automatic parcellation of subcortical structures and estimation of intra-cranial volume (methods S2). Click here for file
