# Supplementary material for: Volumetric associations between uncinate fasciculus, amygdala, and trait anxiety
Source: BMC Neurosci. 2012 Jan 4;13:4. doi: 10.1186/1471-2202-13-4 (PMC3398321; doi:10.1186/1471-2202-13-4)
Supplement: Additional file 1 — Supplemental Results. Distribution of trait anxiety across subjects (figure S1); Scatter plots for associations between trait anxiety and white matter/grey matter volumes of interest (figure S2); Associations of mean fractional anisotropy across the uncinate fasciculus with trait anxiety and amygdala volume (table S1); Associations of uncinate fasciculus, amygdala and hippocampus volume with depression, anxiety sensitivity, behavioral inhibition, and neuroticism (table S2); Associations between the remaining grey matter volumes and trait anxiety (table S3); Absolute and relative volumes of all examined white matter and grey matter structures (table S4). [file 1471-2202-13-4-S1.DOCX]

**Supplemental Results**

**Figure S1**

**Distribution of trait anxiety across subjects**


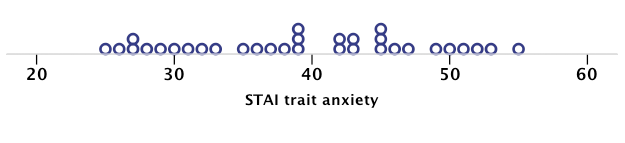


**Figure S2**

**Scatter plots for associations between trait anxiety and WM/GM volumes of interest**

**
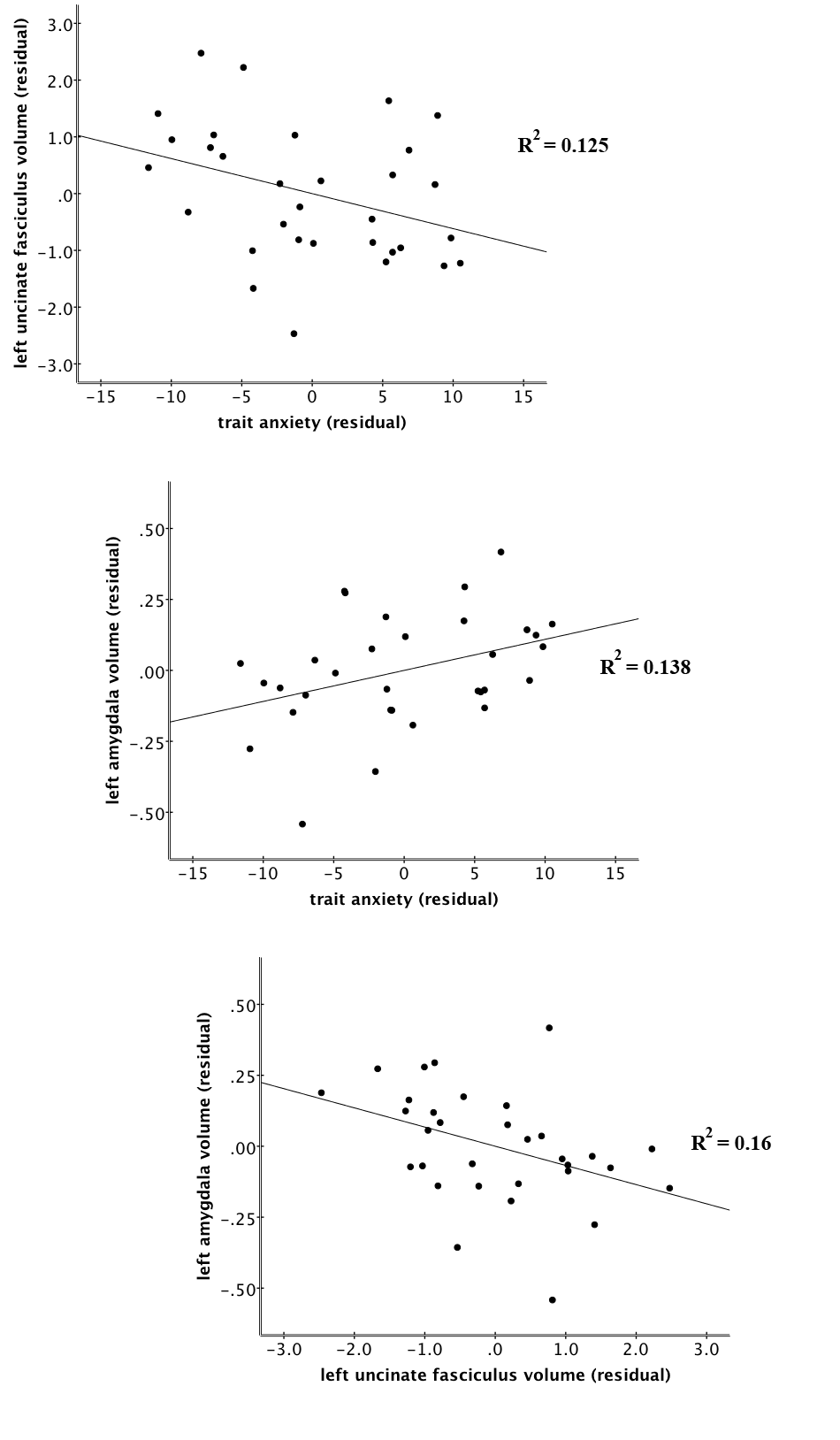
Table S1**

**Associations of mean fractional anisotropy across the uncinate fasciculus with trait anxiety and amygdala volume**

|  | mean FA | |  | correlation ^a^ with | |
| --- | --- | --- | --- | --- | --- |
|  | absolute | relative |  | trait anxiety | amygdala volume |
| left uncinate fasciculus | 0.422  *SD* = 0.021 | 0.884  *SD* = 0.039 |  | -0.32 (0.090) | -0.30 ^b^  (0.111) |
| right uncinate fasciculus | 0.422  *SD* = 0.017 | 0.882  *SD* = 0.033 |  | -0.15 (0.446) | 0.06 ^c^  (0.770) |

^a^ using partial correlations of relative mean FA (tract mean FA divided by global mean FA) with age, sex and depression as covariates of no interest, shown are *r*-values (*p*-values in brackets)

^b^ correlation with left amygdala volume

^c^ correlation with right amygdala volume

FA: fractional anisotropy, SD: standard deviation

Note: For the uncinate fasciculus, volume and mean FA were positively correlated (left hemisphere: *r* = 0.44, *p* < 0.05; right hemisphere: *r* = 0.51, *p* < 0.01). These correlations were assessed using respective relative values (controlling for global effects) with age and sex as covariates of no interest.

**Table S2**

**Associations of uncinate fasciculus, amygdala and hippocampus volume with depression, anxiety sensitivity, behavioral inhibition, and neuroticism**

a) using partial correlations with age and sex as covariates of no interest, shown are *r*-values (*p*-values in brackets)

|  | depression | anxiety sensitivity | behavioral inhibition | neuroticism |
| --- | --- | --- | --- | --- |
| left uncinate fasciculus | -0.02 (0.927) | 0.21 (0.277) | -0.21 (0.275) | -0.14 (0.474) |
| left amygdala | 0.16 (0.412) | -0.10 (0.612) | 0.16 (0.387) | 0.08 (0.663) |
| left hippocampus | 0.07 (0.711) | -0.04 (0.825) | 0.05 (0.782) | 0.07 (0.713) |
|  |  |  |  |  |
| right uncinate fasciculus | -0.06 (0.734) | 0.03 (0.884) | 0.09 (0.649) | -0.05 (0.810) |
| right amygdala | 0.01 (0.961) | -0.11 (0.546) | 0.11 (0.573) | -0.04 (0.836) |
| right hippocampus | -0.06 (0.764) | -0.18 (0.344) | 0.15 (0.439) | 0.02 (0.899) |

Note: To make these partial correlations more comparable to those applied in the main manuscript, we also assessed respective correlations with anxiety sensitivity, behavioral inhibition and neuroticism using depression as additional covariate of no interest. Notably, there was no meaningful change for any of the correlations (i.e., direction of effect was preserved with no significant or marginally significant *r*-value).

b) using partial correlations with age, sex and trait anxiety as covariates of no interest, shown are *r*-values (*p*-values in brackets)

|  | depression | anxiety sensitivity | behavioral inhibition | neuroticism |
| --- | --- | --- | --- | --- |
| left uncinate fasciculus | 0.21 (0.272) | 0.33 (0.082) | -0.02 (0.901) | 0.11 (0.564) |
| left amygdala | -0.11 (0.577) | -0.25 (0.191) | -0.12 (0.534) | -0.30 (0.109) |
| left hippocampus | -0.16 (0.420) | -0.16 (0.421) | -0.20 (0.294) | -0.23 (0.228) |
|  |  |  |  |  |
| right uncinate fasciculus | 0.04 (0.846) | 0.08 (0.672) | 0.25 (0.195) | 0.10 (0.619) |
| right amygdala | -0.17 (0.388) | -0.20 (0.293) | -0.05 (0.787) | -0.30 (0.114) |
| right hippocampus | -0.33 (0.083) | -0.31 (0.104) | -0.07 (0.699) | -0.30 (0.109) |

**Table S3**

**Associations between the remaining grey matter volumes and trait anxiety**

| hemisphere | structure | association with  trait anxiety ^a^ |
| --- | --- | --- |
| left | thalamus | *0.36 (0.058)* |
|  | putamen | 0.19 (0.336) |
|  | pallidum | 0.16 (0.408) |
|  | nucleus accumbens | 0.20 (0.300) |
|  |  |  |
| right | thalamus | 0.20 (0.296) |
|  | putamen | 0.15 (0.440) |
|  | pallidum | 0.15 (0.427) |
|  | nucleus accumbens | 0.21 (0.282) |

^a^ using partial correlations with age, sex, and depression as covariates of no interest, shown are *r*-values (*p*-values in brackets, significant results indicated in bold, marginally significant results indicated in italics).

^b^ one-tailed

**Table S4**

**Absolute and relative volumes of all examined white matter and grey matter structures**

(mean with standard deviation in brackets)

|  |  |  | left | | right | |
| --- | --- | --- | --- | --- | --- | --- |
|  |  |  |  | |  | |
|  |  |  |  |  |  |  |
| brain tissue | structure |  | absolute ^b^ | *relative ^c^* | absolute ^b^ | *relative ^c^* |
|  |  |  |  |  |  |  |
| *white matter* | UF |  | 3.74 (1.58) | *3.44 (1.27)* | 3.87 (0.98) | *3.58 (0.76)* |
|  | IFOF |  | 8.16 (2.81) | *7.64 (2.60)* | 7.82 (2.65) | *7.25 (2.36)* |
|  |  |  |  |  |  |  |
| *grey matter* | AMYG |  | 1.75 (0.25) | *1.64 (0.21)* | 1.86 (0.24) | *1.74 (0.23)* |
|  | HIPP |  | 4.36 (0.44) | *4.07 (0.38)* | 4.43 (0.49) | *4.14 (0.46)* |
|  | THAL |  | 7.28 (0.62) | *6.80 (0.57)* | 7.11 (0.51) | *6.65 (0.56)* |
|  | CAUD |  | 3.95 (0.51) | *3.68 (0.47)* | 4.02 (0.51) | *3.75 (0.42)* |
|  | PUTA |  | 6.37 (0.68) | *5.95 (0.63)* | 6.02 (0.59) | *5.63 (0.62)* |
|  | PALL |  | 1.81 (0.21) | *1.69 (0.18)* | 1.69 (0.19) | *1.58 (0.17)* |
|  | NACC |  | 0.73 (0.11) | *0.69 (0.11)* | 0.78 (0.11) | *0.73 (0.10)* |

^b^ in ml

^c^ in ml (divided by intracranial volume, subsequently multiplied by 1000)

UF: uncinate fasciculus, IFOF: inferior fronto-occipital fasciculus, AMYG: amygdala, HIPP: hippocampus, THAL: thalamus, CAUD: caudate nucleus, PUTA: putamen, PALL: pallidum, NACC: nucleus accumbens
