# Supplementary material for: Volumetric associations between uncinate fasciculus, amygdala, and trait anxiety
Source: BMC Neurosci. 2012 Jan 4;13:4. doi: 10.1186/1471-2202-13-4 (PMC3398321; doi:10.1186/1471-2202-13-4)
Supplement: Additional file 2 — Supplemental Methods. Diffusion tensor imaging data preprocessing and tractography (methods S1); Automatic parcellation of subcortical structures and estimation of intra-cranial volume (methods S2). [file 1471-2202-13-4-S2.DOC]

**Supplemental Methods**

**Methods S1**

*Diffusion tensor imaging: data preprocessing and fiber tractography (methodological description, also described previously [1])*

Preprocessing was done with FMRIB Software Library (FSL) Version 4.1.8 [2] ([www.fmrib.ox.ac.uk/fsl](http://www.fmrib.ox.ac.uk/fsl)) and comprised the following steps: 1) segregation of brain tissue from non-brain tissue using the Brain Extraction Tool [3]; 2) Eddy current and head movement correction using EDDYCORRECT from FMRIB’s Diffusion Toolbox [2]; 3) rotation of the gradients according to the corrected parameters from step 2); 4) local fitting of diffusion tensors and construction of individual FA maps using DTIFIT from FMRIB’s Diffusion Toolbox [2].

For fiber tracking, Diffusion Toolkit 0.6.1 and TrackVis 0.5.1 were used [4] ([www.trackvis.org](http://www.trackvis.org/)). The preprocessed data from FSL were further processed with Diffusion Toolkit. For each subject, the diffusion tensors were estimated according to the corrected gradients. Deterministic fiber tracking was performed with the “brute-force” approach [5], an automatic procedure commonly used to reconstruct fibers across the whole WM by tracking fibers from each voxel in the brain. The fiber assignment continuous tracking (FACT) algorithm [6] was used. Accordingly, fibers were reconstructed by TrackVis along the principal eigenvector of each voxel’s diffusion tensor. Tracking termination criteria were angle > 45° and FA < 0.2 [7] (individual FA map derived from FSL’s DTIFIT was used as mask image in Diffusion Toolkit). Fiber tracking was performed successively in each subject’s native space. Color-coded FA maps derived from the principal eigenvector of the diffusion tensor in each voxel were used for region-of-interest (ROI) drawing in TrackVis. ROIs were drawn large-sized to include the entirety of the tract of interest and avoid false-negative fibers [8] (**see also figure 2**). All fiber tracts were obtained through a two-ROI approach (seed ROI and target ROI) with logical AND concatenation [9, 10] of the two ROIs, such that only fibers that passed both ROIs were included in the reconstructed tract. Obviously spurious fibers were removed from the fiber tract by using an additional avoidance ROI (logical NOT operation) [9]. For the UF, both the seed and the target ROI was placed in the same coronal slice where the anterior-posterior fibers (coded in green) of the frontal and the temporal lobe were visible at the most posterior point (**see figure 2A** for illustration of the ROI placement and tractography examples for the UF, see also [9]). For the IFOF, the seed ROI was placed in the occipital lobe according to Wakana and colleagues [9]. The target ROI was placed at the densest portion of the fiber bundle projecting anteriorly (coded in green, anterior floor of the external capsule [10]), typically located in the coronal slice that dissects the middle of the corpus callosum body (**see figure 2B** for illustration of the ROI placement and tractography examples for the IFOF). Each tract was reconstructed in both hemispheres, and tracking was randomly performed either first in the left or in the right hemisphere in each subject. After tractography, each individual tract was visually inspected for plausibility with regard to its structure based on general anatomical knowledge and previously published tractography studies [9-11]. For each tract, any voxel touched by a fiber was counted by TrackVis. As such, volume values were obtained by accumulating all voxels belonging to the respective tract.

**Methods S2**

*Automatic parcellation of subcortical structures and estimation of intra-cranial volume*

Volumetric segmentation was performed with the Freesurfer image analysis suite (Version 5.1.0), which is documented and freely available for download online (<http://surfer.nmr.mgh.harvard.edu/>). The technical details of these procedures are described in prior publications [12-23]). Briefly, this processing includes motion correction and averaging of multiple volumetric T1-weighted images (when more than one is available), removal of non-brain tissue using a hybrid watershed/surface deformation procedure [19], automated Talairach transformation, segmentation of the subcortical white matter and deep gray matter volumetric structures (including amygdala, hippocampus, thalamus, caudate, putamen, pallidum, nucleus accumbens, ventricles) [20, 21] intensity normalization [24], tessellation of the gray matter white matter boundary, automated topology correction [22, 25], and surface deformation following intensity gradients to optimally place the gray/white and gray/cerebrospinal fluid borders at the location where the greatest shift in intensity defines the transition to the other tissue class [12, 13, 23]. Freesurfer morphometric procedures have been demonstrated to show good test-retest reliability across scanner manufacturers and across field strengths [17].

The procedure for intra-cranial volume estimation automatically assigns a neuroanatomical label to each voxel in the T1-weighted scan, a label that is based on probabilistic information automatically estimated from a manually labeled training set [26]. The technique has previously been shown to be comparable in accuracy to manual labeling [26,27]. Intra-cranial volume was calculated by the use of an atlas-based normalization procedure, where the atlas-scaling factor is used as a proxy for intra-cranial volume. It has been shown that this estimated intra-cranial volume correlates highly with manually derived measurements of intra-cranial volume [27].

**References**

1. Baur V, Bruhl AB, Herwig U, Eberle T, Rufer M, Delsignore A, Jancke L, Hanggi J: **Evidence of frontotemporal structural hypoconnectivity in social anxiety disorder: A quantitative fiber tractography study**. *Hum Brain Mapp*, in press.

2. Smith SM, Jenkinson M, Woolrich MW, Beckmann CF, Behrens TE, Johansen-Berg H, Bannister PR, De Luca M, Drobnjak I, Flitney DE *et al*: **Advances in functional and structural MR image analysis and implementation as FSL**. *Neuroimage* 2004, **23 Suppl 1**:S208-219.

3. Smith SM: **Fast robust automated brain extraction**. *Hum Brain Mapp* 2002, **17**(3):143-155.

4. Wang R, Benner T, Sorensen AG, Wedeen VJ: **Diffusion Toolkit: A software package for diffusion imaging data processing and tractography**. *Proc Intl Soc Mag Reson Med* 2007, **15**:3720.

5. Huang H, Zhang J, van Zijl PC, Mori S: **Analysis of noise effects on DTI-based tractography using the brute-force and multi-ROI approach**. *Magn Reson Med* 2004, **52**(3):559-565.

6. Mori S, Crain BJ, Chacko VP, van Zijl PC: **Three-dimensional tracking of axonal projections in the brain by magnetic resonance imaging**. *Ann Neurol* 1999, **45**(2):265-269.

7. Mori S, van Zijl PC: **Fiber tracking: principles and strategies - a technical review**. *NMR Biomed* 2002, **15**(7-8):468-480.

8. Yasmin H, Aoki S, Abe O, Nakata Y, Hayashi N, Masutani Y, Goto M, Ohtomo K: **Tract-specific analysis of white matter pathways in healthy subjects: a pilot study using diffusion tensor MRI**. *Neuroradiology* 2009, **51**(12):831-840.

9. Wakana S, Caprihan A, Panzenboeck MM, Fallon JH, Perry M, Gollub RL, Hua K, Zhang J, Jiang H, Dubey P *et al*: **Reproducibility of quantitative tractography methods applied to cerebral white matter**. *Neuroimage* 2007, **36**(3):630-644.

10. Catani M, Howard RJ, Pajevic S, Jones DK: **Virtual in vivo interactive dissection of white matter fasciculi in the human brain**. *Neuroimage* 2002, **17**(1):77-94.

11. Mori S, Kaufmann WE, Davatzikos C, Stieltjes B, Amodei L, Fredericksen K, Pearlson GD, Melhem ER, Solaiyappan M, Raymond GV *et al*: **Imaging cortical association tracts in the human brain using diffusion-tensor-based axonal tracking**. *Magn Reson Med* 2002, **47**(2):215-223.

12. Fischl B, Dale AM: **Measuring the thickness of the human cerebral cortex from magnetic resonance images**. *Proc Natl Acad Sci U S A* 2000, **97**(20):11050-11055.

13. Dale AM, Fischl B, Sereno MI: **Cortical surface-based analysis. I. Segmentation and surface reconstruction**. *Neuroimage* 1999, **9**(2):179-194.

14. Fischl B, Sereno MI, Dale AM: **Cortical surface-based analysis. II: Inflation, flattening, and a surface-based coordinate system**. *Neuroimage* 1999, **9**(2):195-207.

15. Fischl B, Sereno MI, Tootell RB, Dale AM: **High-resolution intersubject averaging and a coordinate system for the cortical surface**. *Hum Brain Mapp* 1999, **8**(4):272-284.

16. Jovicich J, Czanner S, Greve D, Haley E, van der Kouwe A, Gollub R, Kennedy D, Schmitt F, Brown G, Macfall J *et al*: **Reliability in multi-site structural MRI studies: effects of gradient non-linearity correction on phantom and human data**. *Neuroimage* 2006, **30**(2):436-443.

17. Han X, Jovicich J, Salat D, van der Kouwe A, Quinn B, Czanner S, Busa E, Pacheco J, Albert M, Killiany R *et al*: **Reliability of MRI-derived measurements of human cerebral cortical thickness: the effects of field strength, scanner upgrade and manufacturer**. *Neuroimage* 2006, **32**(1):180-194.

18. Fischl B, van der Kouwe A, Destrieux C, Halgren E, Segonne F, Salat DH, Busa E, Seidman LJ, Goldstein J, Kennedy D *et al*: **Automatically parcellating the human cerebral cortex**. *Cereb Cortex* 2004, **14**(1):11-22.

19. Segonne F, Dale AM, Busa E, Glessner M, Salat D, Hahn HK, Fischl B: **A hybrid approach to the skull stripping problem in MRI**. *Neuroimage* 2004, **22**(3):1060-1075.

20. Fischl B, Salat DH, van der Kouwe AJ, Makris N, Segonne F, Quinn BT, Dale AM: **Sequence-independent segmentation of magnetic resonance images**. *Neuroimage* 2004, **23 Suppl 1**:S69-84.

21. Fischl B, Salat DH, Busa E, Albert M, Dieterich M, Haselgrove C, van der Kouwe A, Killiany R, Kennedy D, Klaveness S *et al*: **Whole brain segmentation: automated labeling of neuroanatomical structures in the human brain**. *Neuron* 2002, **33**(3):341-355.

22. Fischl B, Liu A, Dale AM: **Automated manifold surgery: constructing geometrically accurate and topologically correct models of the human cerebral cortex**. *IEEE Trans Med Imaging* 2001, **20**(1):70-80.

23. Dale AM, Sereno MI: **Improved localization of cortical activity by combining EEG and MEG with MRI cortical surface reconstruction: a linear approach**. *J Cogn Neurosci* 1993, **5**:162-176.

24. Sled JG, Zijdenbos AP, Evans AC: **A nonparametric method for automatic correction of intensity nonuniformity in MRI data**. *IEEE Trans Med Imaging* 1998, **17**(1):87-97.

25. Segonne F, Pacheco J, Fischl B: **Geometrically accurate topology-correction of cortical surfaces using nonseparating loops**. *IEEE Trans Med Imaging* 2007, **26**(4):518-529.

26. Fischl B, Salat DH, Busa E, Albert M, Dieterich M, Haselgrove C, van der Kouwe A, Killiany R, Kennedy D, Klaveness S *et al*: **Whole Brain Segmentation: Automated Labeling of Neuroanatomical Structures in the Human Brain**. *Neuron* 2002, **33**(3):341-355.

27. Buckner RL, Head D, Parker J, Fotenos AF, Marcus D, Morris JC, Snyder AZ: **A unified approach for morphometric and functional data analysis in young, old, and demented adults using automated atlas-based head size normalization: reliability and validation against manual measurement of total intracranial volume**. *NeuroImage* 2004, **23**(2):724-738.
